# Building complex carbon skeletons with ethynyl[2.2]paracyclophanes

**DOI:** 10.3762/bjoc.10.209

**Published:** 2014-08-27

**Authors:** Ina Dix, Lidija Bondarenko, Peter G Jones, Thomas Oeser, Henning Hopf

**Affiliations:** 1 Institut für Organische Chemie, Technische Universität Braunschweig, Hagenring 30, D-38106 Braunschweig, Germany, Fax: (+49)531-391-5388; 2 Institut für Anorganische und Analytische Chemie, Technische Universität Braunschweig, Postfach 3329, D-38106 Braunschweig, Germany; 3 Organisch-Chemisches Institut der Universität Heidelberg Im Neuenheimer Feld 270, D-69120 Heidelberg, Germany, Fax: (+49) 6221-544205

**Keywords:** carbon-rich molecules, complex carbon scaffolds, cyclophanes, Glaser coupling, multibridged cyclophanes, X-ray analysis

## Abstract

Ethynyl[2.2]paracyclophanes are shown to be useful substrates for the preparation of complex, highly unsaturated carbon frameworks. Thus both the pseudo-*geminal*- **2** and the pseudo-*ortho*-diethynylcyclophane **4** can be dimerized by Glaser coupling to the respective dimers **9**/**10** and **11**/**12**. Whereas the former isomer pair could not be separated so far, the latter provided the pure diastereomers after extensive column chromatography/recrystallization. Isomer **11** is chiral and could be separated on a column impregnated with cellulose tris(3,5-dimethylphenyl)carbamate. The bridge-extended cyclophane precursor **18** furnished the ring-enlarged cyclophanes **19** and **20** on Glaser–Hay coupling. Cross-coupling of **4** and the planar building block 1,2-diethynylbenzene (**1**) yielded the chiral hetero dimer **22** as the main product. An attempt to prepare the biphenylenophane **27** from the triacetylene **24** by CpCo(CO)_2_-catalyzed cycloisomerization resulted in the formation of the cyclobutadiene Co-complex **26**. Besides by their usual spectroscopic and analytical data, the new cyclophanes **11**, **12**, **19**, **20**, **22**, and **26** were characterized by X-ray structural analysis.

## Introduction

Several years ago we described the preparation of various ethynyl[2.2]paracyclophanes and suggested that these compounds could be developed into useful building blocks for the construction of larger, stereochemically complex carbon frameworks (scaffolds) [2]. This prediction is clearly becoming reality, as shown by the growing use of ethynylcyclophanes as substrates for the preparation of carbon-rich organic compounds [3–6].

**Scheme 1:**
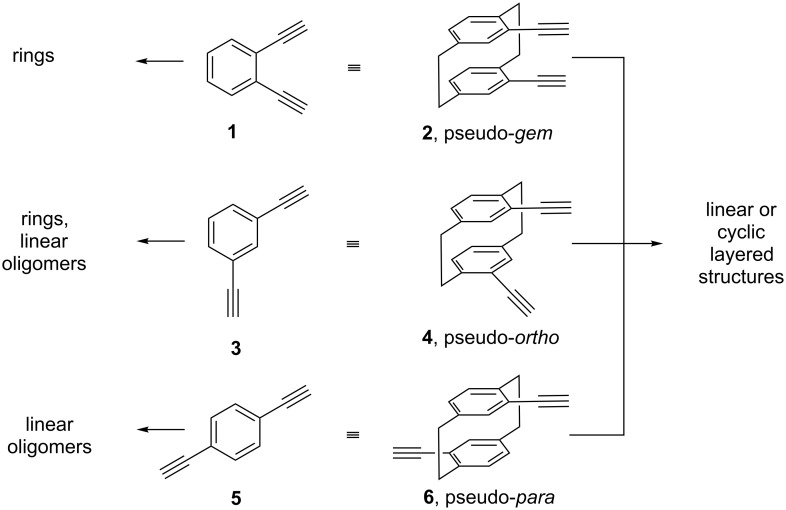
Planar and layered ethynyl aromatics as building blocks for extended aromatic structures.

The use of ethynylaromatics for the synthesis of “extended aromatic compounds” is anything but new, as indicated by three of the smallest building blocks applied for this purpose: 1,2- (**1**), 1,3- (**3**), and 1,4-diethynylbenzene (**5**) (Scheme 1).

These simple, flat molecules have been used extensively for the deliberate construction of larger polyaromatic hydrocarbons employing classical (e.g., Glaser coupling and its variants) or modern acetylenic coupling reactions (e.g., the Sonogashira coupling) [7]. For the smaller oligomers (dimers, trimers) the *ortho*-isomer with its opening angle of 60° between the ethynyl functions leads preferentially to (mono)cyclic hydrocarbons. For the *meta*-compound **3** we can expect both cyclic and acyclic (linear) products, and when the two ethynyl moieties are anchored in *para*-position, **5**, the lower oligomers can no longer be cyclic because they would be too highly strained.

When two ethynyl groups are placed into the benzene rings of [2.2]paracyclophane, the situation changes. In a strict sense the analog of 1,2-diethynylbenzene (**1**) is 4,5-diethynyl[2.2]paracyclophane, i.e., the hydrocarbon with two ethynyl groups in vicinal position in the same ring [2–3]. If, however, our target molecules are to have the two triple bonds in different benzene rings, the pseudo-*gem*-diethynyl[2.2]paracyclophane **2** is the analog of **1** (Scheme 1). Analogously, phane hydrocarbons **4** and **6** correspond to **3** and **5**. Clearly, in all cases employing **2**, **4**, and **6** as building blocks, the final structures will be layered.

**Scheme 2:**
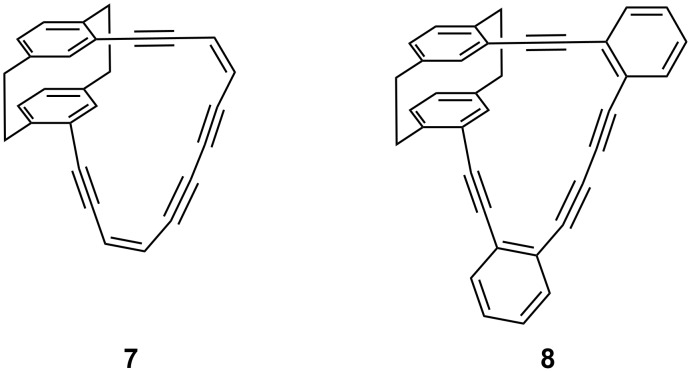
Previous coupling experiments with pseudo-*ortho*-diethynyl[2.2]paracyclophane **4**.

We have already used the pseudo-*ortho*-isomer **4** in two cases describing the preparation and structural properties of the (chiral) tetraynes **7** and **8** (Scheme 2) [8].

In the present contribution we have extended these studies, employing **2** and **4** as building blocks. The chemistry of [*m*.*n*]paracyclophanes with (completely or partially) unsaturated molecular bridges has been poorly investigated, leaving much scope for further studies.

## Results and Discussion

**Scheme 3:**
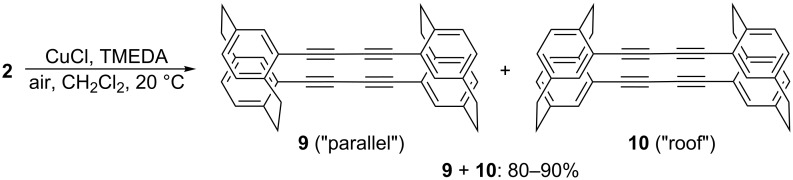
Glaser coupling of pseudo-*gem*-diethynyl[2.2]paracyclophane **2**.

The oxidative dimerization (Glaser coupling) of the achiral hydrocarbon **2** took place effortlessly and in high yield (Scheme 3).

However, we have been unable so far to determine the exact structure of the isolated dimer. As shown in Scheme 3, in principle, two different dimers of **2** could be formed: one in which the ethano bridges are arranged in a parallel fashion (**9**), and another one in which they point towards each other (“roof” isomer **10**). We could not separate the two diastereomers chromatographically (neither by hplc or tlc) so far, nor do the proton and carbon spectra provide conclusive structural information. Our proposal of two types of dimers results, firstly, from the spectra of the dimers generated by Glaser coupling of 4-ethynyl[2.2]paracyclophane and secondly, from the dimerization results with the pseudo-*ortho* compound **4** described below. NMR analysis proved unambiguously that two diastereomers are generated (as expected) by the oxidative dimerization of the mono ethynyl derivative [9], but assignment of the various spectra to specific stereoisomers remains an open question, and will only be possible after the resolution of the 4-ethynyl[2.2]paracyclophane, determination of its absolute configuration, and oxidative dimerization of an enantiopure sample.

**Scheme 4:**
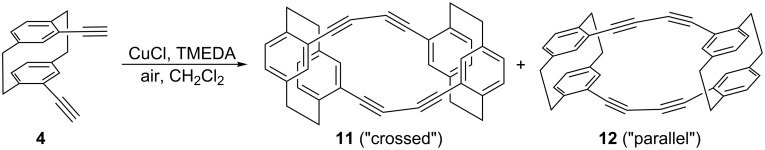
Glaser coupling of pseudo-*ortho*-diethynyl[2.2]paracyclophane, **4**.

Glaser coupling of racemic **4** at room temperature yielded a mixture of diastereomeric dimers in good yield (67%) under the conditions shown in Scheme 4. Their gross structures follow from the spectroscopic and analytical data summarized in the Supporting Information File 1.

**Figure 1:**
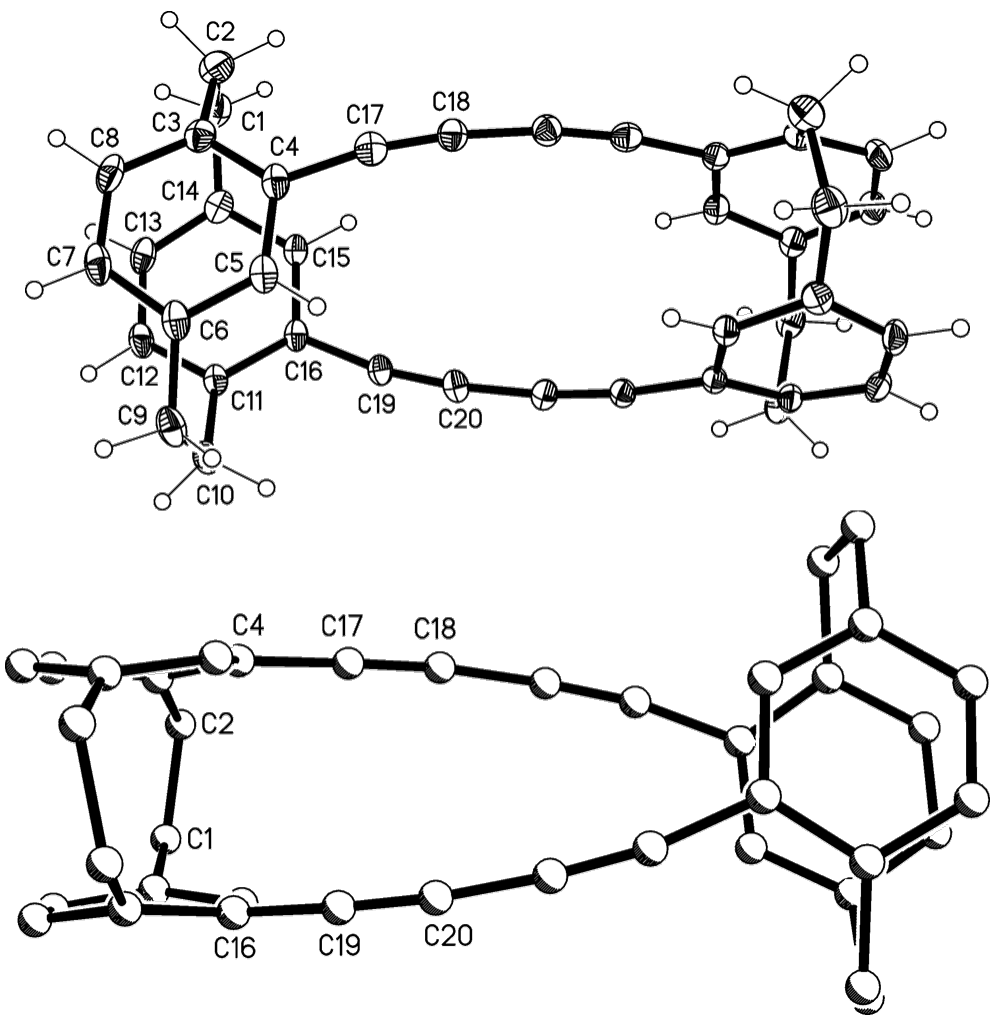
Above: The molecule of compound **11** in the crystal; ellipsoids represent 30% probability levels. Only the asymmetric unit is numbered. Below: Alternative view direction (arbitrary radii, without H atoms) showing the “crossed” geometry via the additional bridges.

The two very poorly soluble hydrocarbons were separated by extensive column chromatography which – albeit in poor yield – finally furnished the analytically pure dimers **11** and **12**, the former eluting more rapidly from the chromatography column. Both samples could be recrystallized to provide single crystals suitable for X-ray structural analysis. Figure 1 shows that dimer **11** indeed possesses the “crossed” structure; it crystallizes with imposed twofold symmetry, but the effective (non-crystallographic) symmetry is the unusual *D*_2_ (*222*) with r.m.s. deviation 0.01 Å.

Despite the extra diacetylene bridges, compound **11** preserves the general structural features of [2.2]paracyclophanes, which are strained molecules. We have discussed these in detail in our previous paper [1], and summarize them here as follows: the single bonds in the bridges, C1–C2 and C9–C10, are elongated and the sp^3^ angles at these atoms widened; the sp^2^ angles at the bridgehead atoms C3, C6, C11 and C14 are narrowed; the rings display a flattened boat conformation in which the bridgehead atoms lie ca. 0.12–0.16 Å out of the plane of the other four atoms; these planes are approximately parallel to each other, as are the vectors between the bridgehead atoms, and the non-bonded contacts between bridgehead atoms are necessarily short (2.7–2.8 Å) [4]. One slight exception for **11** is the twist of 5.7° between C3···C6 and C11···C14. The extra acetylenic bridges have little or no clamping effect, with non-bonded distances C4···C15 and C5···C16 of ca. 3.07 Å (this is also the case for the other “double paracyclophane” structures presented here (see below) and will not be mentioned again explicitly). The slight bowing of the extra bridges, with angles at the sp carbon of 170–173°, can be recognized in the Figure; we regard this angle as a “soft” parameter [1]. The angle between the two halves of the molecule, expressed as the interplanar angle between the plane of C4, C5, C7, C8 and its symmetry-equivalent, is 77°, and this is clearly imposed by the “crossed” bridge geometry.

Isomer **11**, a chiral compound, was resolved into its enantiomers on a OD-column impregnated with cellulose tris(3,5-dimethylphenyl) carbamate using hexane/propan-2-ol (9:1) as an eluent and a UV-detector set at 254 nm. A baseline separation was achieved and the two enantiomers had [α]_D_^25^ = −44 ° (*c* 0.375, hexane/propan-2-ol, 9:1) and [α]_D_^25^ = 43 ° (*c* 0.533, hexane/propan-2-ol, 9:1).

**Figure 2:**
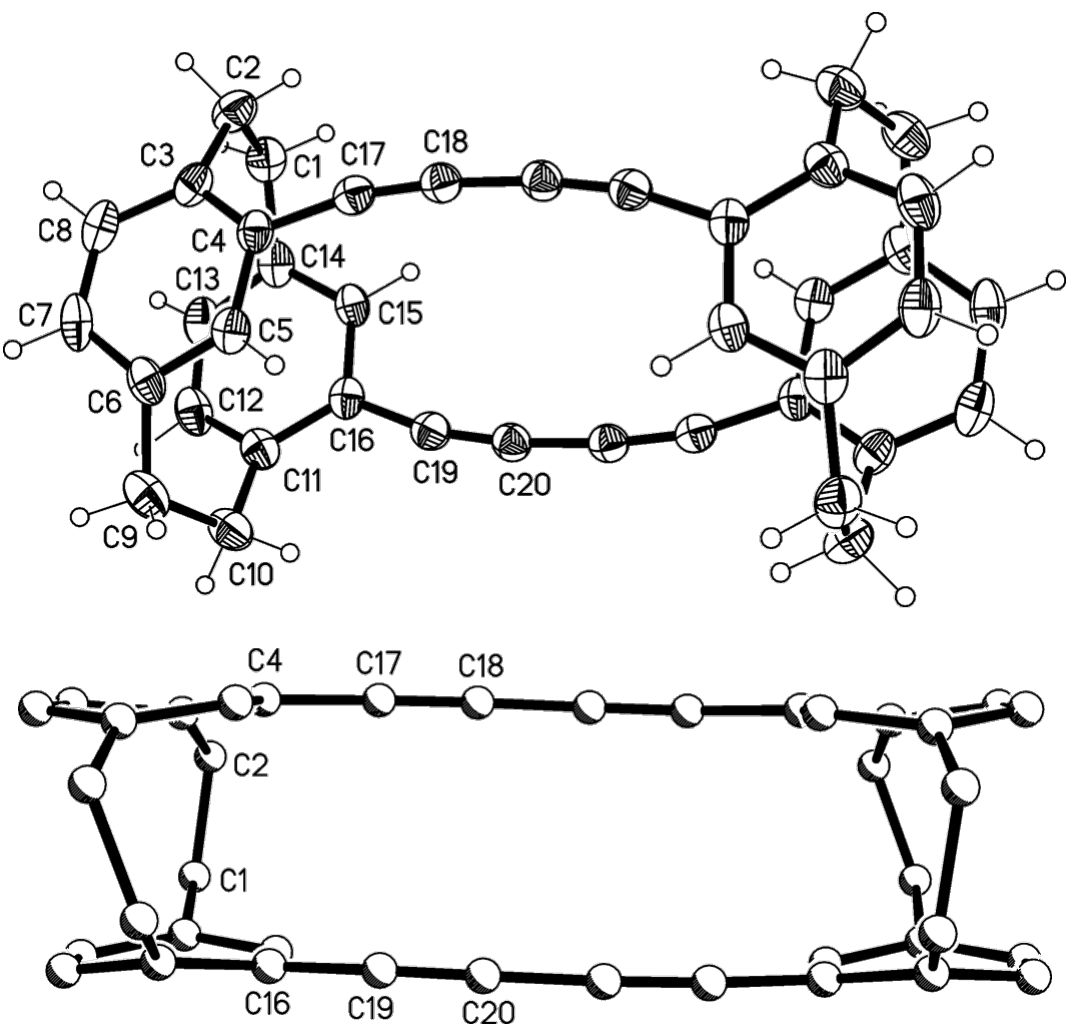
Above: The molecule of compound **12** in the crystal; ellipsoids represent 50% probability levels. Only the asymmetric unit is numbered. Below: Alternative view direction (arbitrary radii, without H atoms) showing the “parallel” geometry via the additional bridges.

The later eluting dimer is the “parallel” hydrocarbon **12** (Figure 2). This compound (Figure 2) crystallizes with imposed inversion symmetry (and is thus achiral), but the effective symmetry is *C*_2h_ (*2*/*m*) with r.m.s.d. 0.14 Å.

Compound **12** displays the “parallel” geometry of the extra bridges, and the two paracyclophane units are indeed exactly parallel to each other by symmetry. The cyclophane rings of the asymmetric unit are twisted by 8.4°. The extra bridges are again bowed, and the angles depart slightly more from 180° (167° at C17 and C19).

**Scheme 5:**
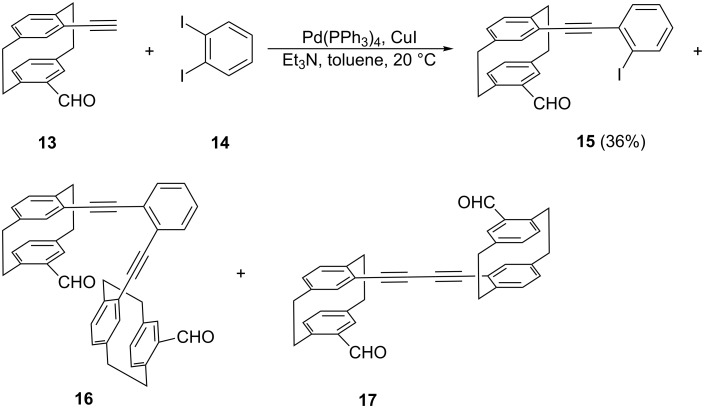
Sonogashira coupling of aldehyde **13** with *ortho*-diiodobenzene (**14**).

For the preparation of “benzologs” of **11** and **12** in which an *ortho*-disubstituted benzene ring has been inserted into one of the butadiyne units, we needed the bisaldehyde **16** (Scheme 5) as the starting material.

Although its synthesis from the previously described ethynylaldehyde **13** [2–3] appears simple, we always obtained complex mixtures of products when **13** and excess 1,2-diiodobenzene (**14**) were subjected to Sonogashira coupling. The main product of this coupling process was the monoaldehyde **15** (i.e., the 1:1-coupling product of **13** and **14**). The desired 2:1-product **16** was always isolated as a side-product together with the dimer of the substrate, dialdehyde **17**. Although these two compounds could be separated on a small scale for analytical purposes (see data in Supporting Information File 1) by extensive column chromatography, for further transformations a **16**/**17** mixture was employed, leaving the ultimate separation/purification to the very end of the synthesis (see Supporting Information File 1).

**Scheme 6:**
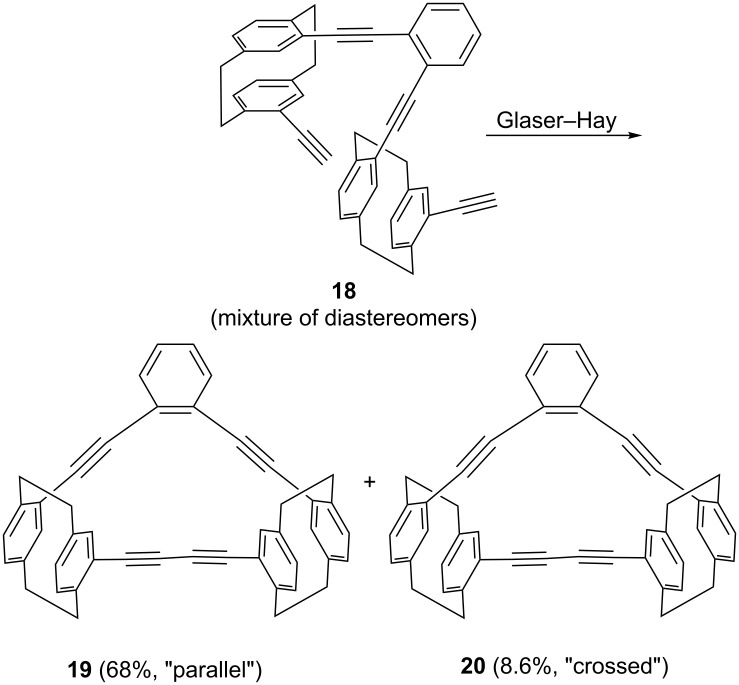
Preparation of benzologs of dimers **11**/**12**.

Having converted **16** by the Bestmann–Ohira transformation [10] into the tetrayne **18**, the stage was set for a final Glaser coupling (Scheme 6).

**Figure 3:**
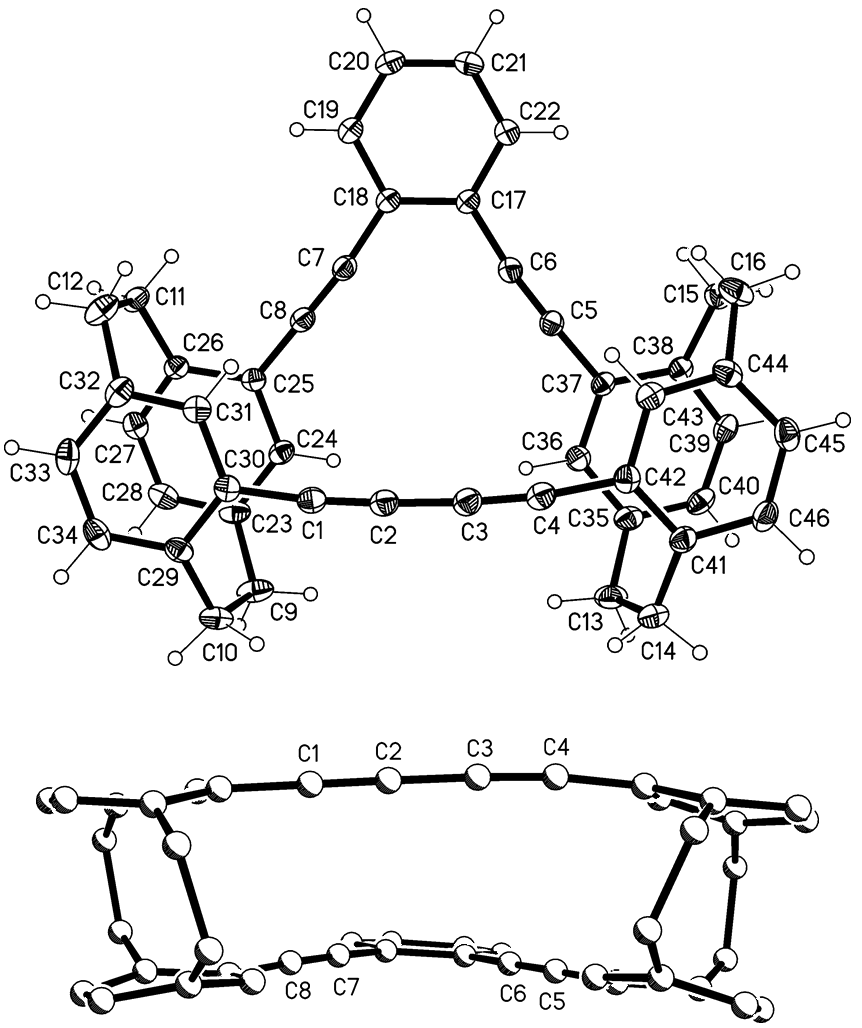
Above: The molecule of compound **19** in the crystal; ellipsoids represent 50% probability levels. Solvent is omitted for clarity. Below: Alternative view direction (arbitrary radii, without H atoms) showing the “parallel” geometry via the additional bridges.

**Figure 4:**
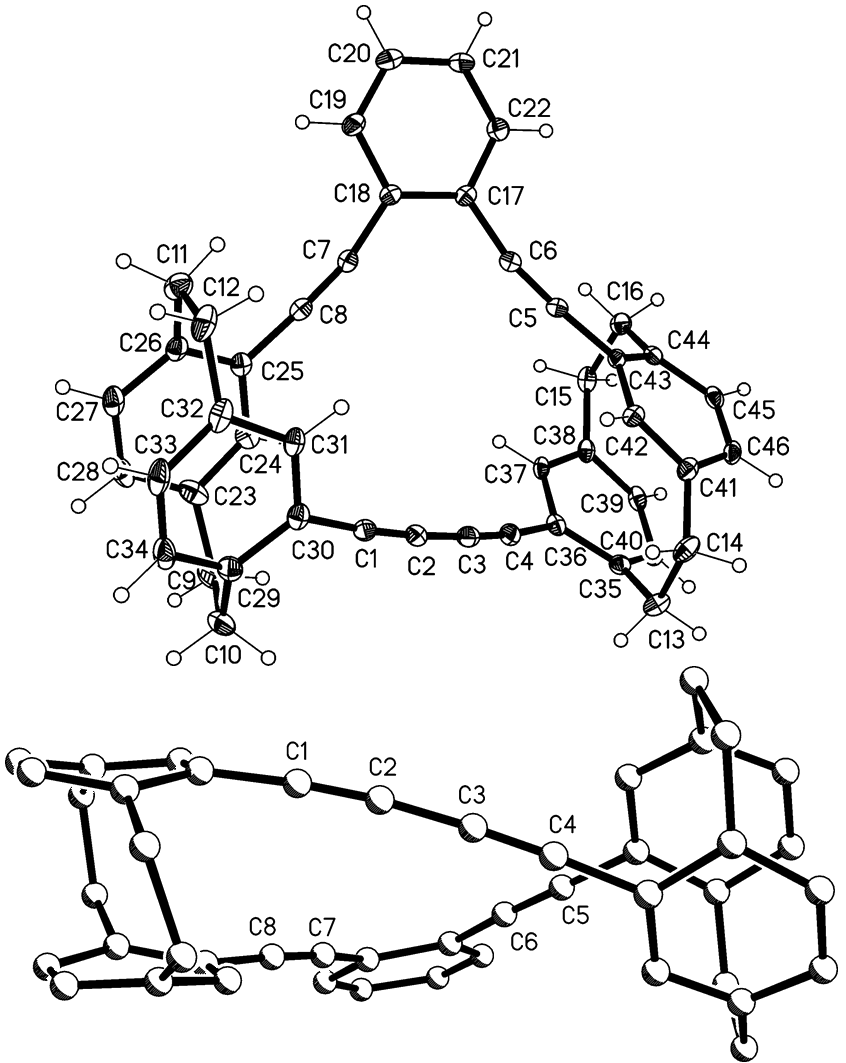
Above: One of the three independent molecules of compound **20** in the crystal; ellipsoids represent 30% probability levels. Solvent is omitted for clarity. Below: Alternative view direction (arbitrary radii, without H atoms) of the same molecule, showing the “crossed” geometry via the additional bridges.

This provided a mixture of the two hydrocarbons **19** and **20** (total yield 78%) which both could be obtained in pure and crystalline form by repeated chromatography and recrystalli-zation. The final structural proof was again provided by single crystal X-ray analysis (Figure 3 and Figure 4).

Compound **19** (Figure 3) has approximate mirror symmetry (r.m.s.d. 0.12 Å). The bridges are “parallel”, although the angle between the two cyclophane units is ca. 16° (the average of the interplanar angles between the two halves across the pseudo-mirror plane). The two paracyclophane units each show the usual patterns of strain; the twist angles are 1.4 and 4.4°. The eight sp angles are reasonably linear, averaging 175°.

The corresponding “crossed” derivative **20** crystallizes with three independent molecules, which are reasonably similar (r.m.s.d. of least-squares fits: molecules 1 and 2, 0.19 Å; molecules 1 and 3, 0.15 Å), but molecular symmetry within the program tolerance was only found for molecule 1, which has *C*_2_ (*2*) symmetry with an r.m.s.d. of 0.22 Å (Figure 4). The molecules show little strain apart from the standard cyclophane features, with small twist angles of 0.9–4.3°. Exceptions are furnished by the sp angles at C6 and C7, which lie in the range 168–170° for all three molecules; all other sp angles are 172–176°. The angles between the cyclophane units are ca. 61, 54, 57° for the three independent molecules.

**Scheme 7:**
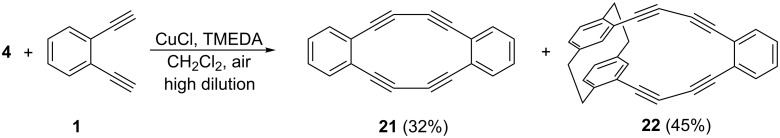
Cross dimerization of **1** and **4**.

In a final Glaser coupling experiment we wished to cross-couple a representative of either the “flat” series **1**, **3**, and **5** with a representative of the “layered” series **2**, **4**, and **6**, and for this purpose chose the coupling between **1** and **4** (Scheme 7).

**Figure 5:**
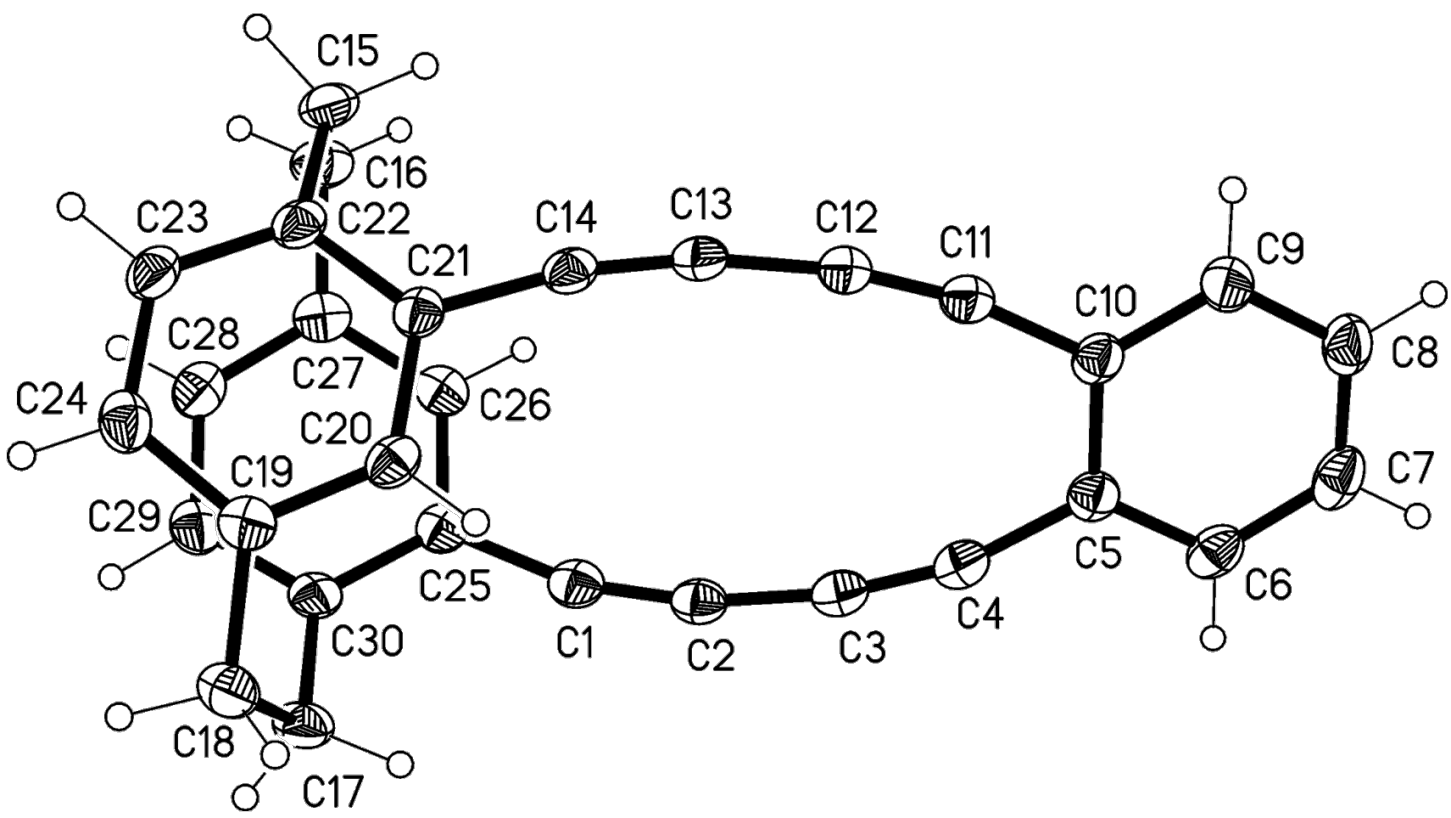
The molecule of compound **22** in the crystal; ellipsoids represent 50% probability levels.

The desired product **22** was indeed isolated as the main product (45%) besides the homo dimer of **1**, the known [11] tetrayne **21** (32%). The unambiguous structural proof for **22** was again provided by single-crystal X-ray analysis (Figure 5); all other analytical and spectroscopic data are collected in the experimental section (see Supporting Information File 1).

The “crossed” derivative **22**, with its relatively short extra acetylenic bridges and with the clamping effect of the ring C5–C10, is reminiscent of the more strained derivatives presented in the previous paper [1]. The cyclophane rings remain parallel, with a moderate twist angle of 7.8°; they subtend interplanar angles of ca. 40° with the benzene ring. The strain is again shown in the “soft” sp angles (165–171°) and to some extent in the slightly lengthened C≡C bonds, 1.21–1.22 Å. Perhaps unexpectedly, the main manifestation of strain is to lengthen the aromatic bond C5–C10 to 1.444(3) Å, equal in length to the formally single bonds such as C4–C5 that link the acetylenic bridges to the ring systems.

**Scheme 8:**
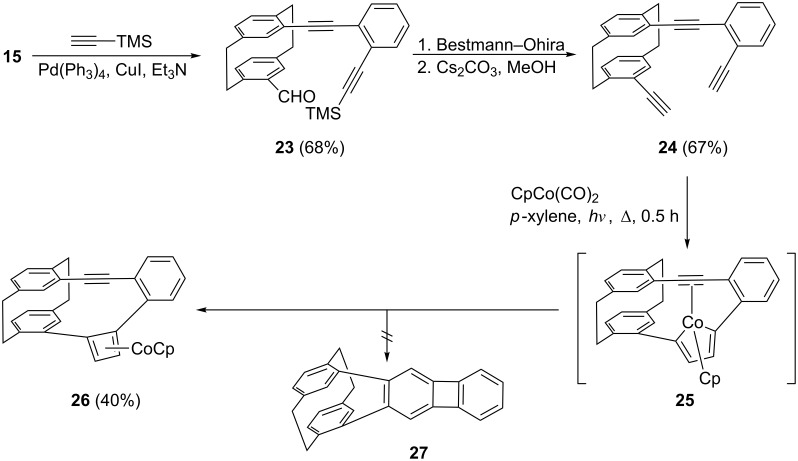
An attempt to prepare a biphenylenophane.

The monoiodide **15**, prepared as described in Scheme 5, offers itself for another coupling/cycloisomerization sequence which, in principle, could provide a hybrid molecule consisting of a [2.2]paracyclophane core and a biphenylene bridge, hydrocarbon **27** (Scheme 8).

To prepare this new (and also chiral) cyclophane system we applied the following pathway. Sonogashira coupling of iodide **15** with trimethylsilylacetylene furnished the TMS-protected aldehyde **23** in good yield. Deprotection and conversion of its formyl function into an ethynyl group by the Bestmann–Ohira protocol took place readily and provided the triacetylene **24**, again in good yield (67%; for the spectroscopic data see Supporting Information File 1). In this intermediate the three triple bonds to be converted into a benzene ring possess only one degree of freedom: the rotation of the non-phane benzene ring around its connecting acetylene group. The cyclotrimerization of three triple bonds under the influence of a cobalt catalyst such as CpCo(CO)_2_ has been observed many times, notably by the Vollhardt group [12].

**Figure 6:**
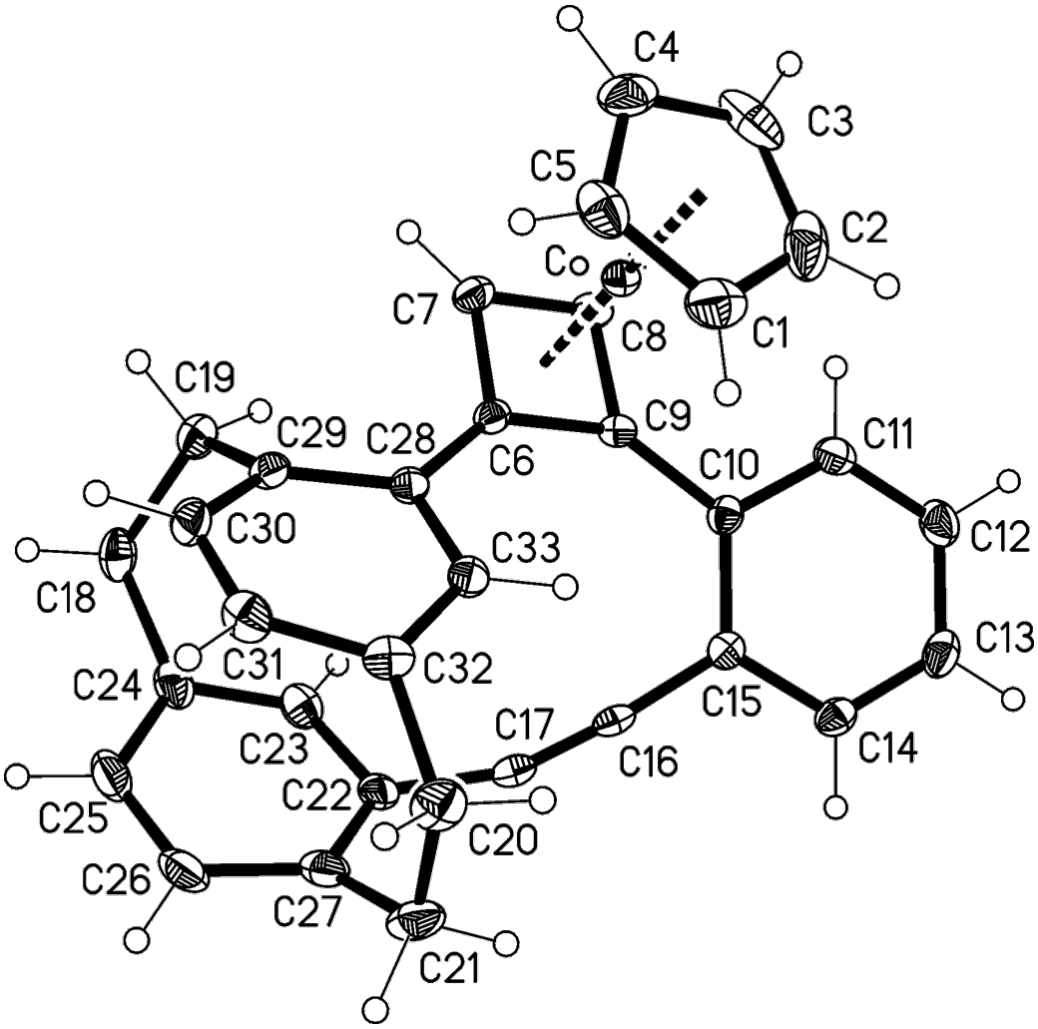
The molecule of compound **26** in the crystal; ellipsoids represent 50% probability levels.

In our case, however, the process is not complete. Rather than yielding the expected biphenylenophane **27**, the reaction stops at the stage of the cyclobutadiene complex **26**, which is isolated in moderate yield (40%, Scheme 8). We propose that the cyclization process begins at the two most accessible ethynyl groups of **24**, generating the cobaltocyclopentadiene intermediate **25**. This cannot proceed to the aromatic ring, since the strain increase associated with the last step is prohibitive. Instead it prefers the isomerization to the isolated CpCo-complex **26**. Compound **26** was identified by its spectroscopic data (see Supporting Information File 1) and also by a single-crystal X-ray analysis. The result is displayed in Figure 6.

The cobalt complex **26** shows essentially normal geometries for the metal center and the cyclophane systems, although the latter show a twist angle of 9.8°; they are tilted by ca. 45° with respect to the aromatic ring C10–C15. There is some evidence of strain in the short bridges between the cyclophanes; thus the bond C6–C9 is at 1.499(2) Å the longest in the four-membered ring, while the angles at C9 and C6 [144.4(2), 140.0(2); ideally bisecting values would be 135°] are widened and that at C17, 162.7(2)°, is narrowed. A similar cyclobutadiene complex has been observed by Vollhardt and his co-workers, in a phenylene synthesis in which a planar triacetylene was subjected to CpCo(CO)_2_-mediated cycloisomerization [13].

## Conclusion

In conclusion, we have considerably extended the range of highly unsaturated carbon scaffolds by using ethynyl[2.2]paracyclophanes as substrates for the generation of new layered frameworks. The above approach is rendered even more attractive by the fact that several of these display interesting stereochemical properties.

## Supporting Information

File 1 Experimental section.
