# Supplementary material for: Building complex carbon skeletons with ethynyl[2.2]paracyclophanes
Source: Beilstein J Org Chem. 2014 Aug 27;10:2013–20. doi: 10.3762/bjoc.10.209 (PMC4168697; doi:10.3762/bjoc.10.209)
Supplement: File 1 — Experimental section. [file Beilstein_J_Org_Chem-10-2013-s001.pdf]

Supporting Information

for

Building complex carbon skeletons with ethynyl[2.2]paracyclophanes

Ina Dix[1], Lidija Bondarenko[1], Peter G. Jones[2], Thomas Oeser[3] and Henning Hopf*[1]

Address: [1]Institut für Organische Chemie, Technische Universität Braunschweig, Hagenring 30, D-38106 Braunschweig (Germany), Fax: (+49)531-391-5388, [2]Institut für Anorganische und Analytische Chemie, Technische Universität Braunschweig, Postfach 3329, D-38106 Braunschweig (Germany) and [3]Organisch-Chemisches Institut der Universität Heidelberg Im Neuenheimer Feld 270, D-69120 Heidelberg (Germany), Fax: (+49) 6221-544205

Email: Henning Hopf* - h.hopf@tu-bs.de; Thomas Oeser - thomas.oeser@oci.uni-heidelberg.de; Peter G. Jones - p.jones@tu-bs.de

*Corresponding author

General: ^{1}H and ^{13}C NMR spectra: *Bruker* AC 200, ^{1}H NMR (200.1 MHz), ^{13}C NMR (50.3 MHz) and *Bruker* DRX-400, ^{1}H NMR (400.1 MHz), ^{13}C NMR (100.6 MHz) spectrometer. Chemical shifts (δ) are expressed in ppm downfield from tetramethylsilane using the residual non-deuterated solvent as internal standard ($CDCl_3$: ^{1}H: δ = 7.26; ^{13}C: δ = 77.00). Coupling constants are expressed in Hz; the degree of substitution of the carbon atoms was determined by employing the DEPT-135 technique. – IR: Nicolet 320 FT-IR spectrometer and Bruker Tensor 27. - UV/Vis: Beckman UV 8452A Diode Array Spectrophotometer. – MS: Finnigan MAT 90 spectrometer (EI, 70 eV). - Melting points: Büchi 510 melting point apparatus, uncorrected. - Anhydrous THF, dichloromethane and Et_2O were distilled from CaH_2 under nitrogen prior to use. – TLC: precoated plastic plates, polygram Sil G/ UV_{254} Macherey-Nagel & Co. (Düren); CC: Silica gel 60 (70-230 mesh) from Merck. Preparative TLC: PLC plates Silica gel 60 $F_{254+366}$, 2 mm, Merck. - 4-Formyl-16-ethynyl[2.2]paracyclophane (**13**) was prepared as described in the literature [1].

Glaser-Hay dimerization of 4,15-diethynyl[2.2]paracyclophane (2): To a solution of diyne **2** (128 mg, 0.5 mmol) in anhydrous dichloromethane (15 mL) was added CuCl (70 mg, 0.7 mmol) and TMEDA (1 mL). The mixture was stirred for 2 h

under dry air ($CaCl_2$-tube); during this time the color changed from green to grey-brown and was accompanied by precipitation. To the reaction mixture was added a solution of dil. aqueous hydrochloric acid and ethanol, and the precipitate removed by filtration. The solid residue was washed with ethanol and ether, and dried in air to give 120 mg (93 %) of a yellow solid, **9**/**10**, which darkened at room temp. within several days; m. p. > 200 °C (decomp.). - ^{1}H NMR (400 MHz in CS_2/C_6D_6): δ = 2.78 (dd, *J* = 10.1, 12.9, 4 H), 2.92 (dd, *J* = 4.0, 13.1 Hz, 2 H), 3.94 (dd, *J* = 4.0, 13.1 Hz, 2 H), 6.12 (dd, *J* = 1.82, 7.8 Hz, 2 H), 6.29 (d, *J* = 7.8 Hz, 2 H), 6.68 ppm (d, *J* = 1.8 Hz, 2 H). - ^{13}C NMR (100 MHz in CS_2/C_6D_6): δ = 34.67 (t), 35.50 (t), 79.91 (s), 83.16 (s), 123.31 (s), 134.18 (d), 134.34 (d), 137.35 (d), 139.64 (s), 143.69 ppm (s). - IR (diamond-ATR): nu(tilde) = 3305 (w), 3037 (w), 3010 (w), 2959 (w), 2930 (m), 2891 (w), 2851 (m), 2205 (w), 2138 (w), 1907 (w), 1584 (m), 1477 (m), 1450 (m), 1434 (m), 1401 (m), 903 (m), 880 (m), 821 (m), 722 (s), 647 (m), 613 (m), 543 cm^{-1} (m). - UV (CH_2Cl_2): λ_{max} (logε) = 229 (4.64), 307 (4.50), 327 nm (4.71). MS(EI): *m/z* (%) = 508 (100) [M^+], 317 (88), 252 (76), 121 (68). – HRMS (EI): calcd. for $C_{40}H_{28}$: 508.2191, found 508.2174.

Glaser-Hay dimerization of 4,16-diethynyl[2.2]paracyclophane (4): A solution diyne **4** (256 mg, 1 mmol) in anhydrous dichloromethane (30 mL) was added to a mixture of CuCl (140 mg, 1.4 mmol) and

TMEDA (2 mL). The reaction mixture was stirred for 2 h under dry air ($CaCl_2$-tube), during this time the color changed from green to grey-brown. For work-up a dilute aqueous solution of HCl in ethanol was added, and the precipitate formed was removed by filtration. The residue was washed with ethanol and ether, and dried to give 170 mg (67%) of an off-white solid, dec. 270-320 °C. The mixture of the poorly soluble dimers **11** and **12** provided the following spectroscopic data: ^1H NMR (200 MHz in $CDCl_3$): δ = 2.70-2.90 (m, 4 H), 3.01-3.14 (q, 4 H), 3.35-3.50 (m, 8 H), 6.53-6.56 (m, 8 H), 7.32 (s, 2 H), 7.36 ppm (s, 2 H). - ^1H NMR (400 MHz in CS_2/C_6D_6): δ = 2.45-2.56 (m, 4 H), 2.74-2.87 (m, 4 H), 3.00-3.18 (m, 4 H), 3.21-3.49 (m, 16 H), 6.20-6.27 (m, 8 H), 7.14 (s, 2 H), 7.21 ppm (s, 2 H). - IR (diamond-ATR): nu (tilde) = 2958 (m), 2192 (w), 2125 (w), 1904 (w), 1482 (m), 1432 (m), 1398 (m), 897 (s), 878 (s), 721 (s), 646 (s), 558 (s) cm^{-1}. - UV (CH_2Cl_2): λ_{max} (lgε) = 230 (4.92), 251 (4.72), 307 (4.47), 346 (2.34), 373 nm (4.34). - MS(EI): *m/z* (%)= 508 (100) [M^+], 491 (20), 477 (20), 252 (100). - HRMS (EI): calcd. for $C_{40}H_{28}$: 508.2191; found 508.2181.

The mixture of isomers **11** and **12** was repeatedly column chromatographed on silica gel with pentane-dichloromethane to yield first the "crossed"

hydrocarbon **11**, which was subsequently recrystallized from toluene, m. p. > 280 °C (decomp.). - ^{1}H NMR (400 MHz, CS_2/C_6D_6): δ = 2.78-2.86 (m, 4 H), 3.07 (m, *J* = 11 Hz, 4 H), 3.32 (m, *J* = 11 Hz, 4 H), 3.38-3.46 (m, 4 H), 6.56 (ps-s, 8 H), 7.36 ppm (s, 4 H). - ^{13}C NMR (100 MHz, CS_2/C_6D_6): δ = 29.7 (t), 33.6 (t), 35.1 (t), 84.8 (s), 124.9 (s), 132.6 (d), 134.7 (d), 137.4 (d), 139.8 (s), 141.9 ppm (s). Isomer **11** was resolved into its enantiomers on a OD-column impregnated with cellulose tris(3,5-dimethylphenyl carbamate, 250 × 20 mm, particle size 5 μm using hexane/propan-2-ol (9:1) as an eluent with a flow rate of 8 mL/min and a UV-detector set at 254 nm: $[\alpha]_D^{25}$ = -44 ° (c = 0.375, hexane/propan-2-ol, 9:1); $[\alpha]_D^{25}$ = 43 ° (c = 0.533, hexane/propan-2-ol, 9:1) [10].

By several crystallizations from dichloromethane the pure "parallel" (less soluble) isomer **12** was obtained. - ^{1}H NMR (400 MHz, CS_2 + C_6D_6): δ = 2.70-2.79 (m, 4 H), 2.97-3.11 (m, 4 H), 3.22-3.39 (m, 8 H), 6.45 (dd, *J* = 1.8, 4.3 Hz, 2 H), 6.48 (d, *J* = 4.3 Hz, 2 H), 6.49 (ps-s, 4 H), 7.26 (d, *J* = 1.7 Hz, 2 H), 7.29 ppm (s, 2 H).

Sonogashira coupling of *ortho*-diiodobenzene (14) with 4-formyl-16-ethynyl-[2.2]para-cyclophane (13): A solution of **13** (760 mg, 2.9 mmol) and 1,2-diiodobenzene

(**14**, 1.3 g, 3.9 mmol) in toluene (90 mL) and triethylamine (25 mL) was carefully degassed by bubbling nitrogen through the solution for 1 h. Subsequently was added tetrakis-triphenylphosphine palladium (0) (120 mg, 0.10 mmol, orange) and copper(I)iodide (100 mg, 0.5 mmol), and the mixture stirred under nitrogen at room temp. for 2 h. The reaction mixture was treated with dilute hydrochloric acid (30 mL), the phases were separated, and the water was extracted once more with dichloromethane. The combined organic solutions were washed with water, aqueous sodium bicarbonate solution and brine, and finally dried ($MgSO_4$). The solvent was removed at reduced pressure and the crude mixture was column chromatographed on 60 g of silica gel. Elution with pentane-dichloromethane (25:75) gave first 4-(2-iodophenylethynyl)-16-formyl[2.2]paracyclophane (**15**) as colorless crystals, (480 mg, 36%), m. p. 152-153 °C. - ^{1}H NMR (200 MHz, $CDCl_3$): δ = 2.78-2.95 (m, 2 H), 3.00-3.19 (m, 2 H), 3.33-3.47 (m, 1 H), 3.75-3.47 (m, 1 H), 3.75-3.88 (m, 1 H), 4.12-4.23 (m, 1 H), 6.46-6.71 (m, 4 H, paracyclophanyl H), 7.00-7.01 (dd, *J* = 0.4, 1.7 Hz, 1 H, paracyclophanyl H), 7.29-7.37 (dt *J* = 1.2, 7.5 Hz, 2 H, phenyl H), 7.53-7.57 (m, 2 H, phenyl H), 7.86-7.91 (dd, *J* = 1.0 , 8.0 Hz, 1 H, paracyclophanyl H), 9.95

ppm (s, 1 H, CHO). - ^{13}C NMR (100 Hz, $CDCl_3$): δ = 33.4 (t), 34.0 (t), 34.0 (t), 34.4 (t), 92.5 (s, acetylene), 95.3 (s, acetylene), 100.6 (s, phenyl-I), 124.4 (s), 127.9 (d), 129.4 (d), 130.1 (s), 132.9 (d), 133.1 (d), 134.0 (d), 135.9 (d), 136.2 (d), 136.5 (s), 138.3 (d), 138.8 (d), 140.1 (s), 140.5 (s), 142.3 (s), 193.0 ppm (d, CHO). – IR (KBr): nu(tilde)= 2927 (m), 2886 (w), 2849 (w), 2205 (w), 1684 (s), 1551 m), 1484 (w), 1462 (m), 1430 (m), 758 (s), 720 (m), 647 (m) cm^{-1}. - UV (EtOH): λ_{max} (lgε) = 218 (4.59), 316 nm (4.26). – MS (EI): *m/z* (%) = 462(22) [M^+], 335 (35), 330 (82), 202 (100). - HRMS (EI): calcd. for $C_{25}H_{19}IO$ 462.0481; found 462.0467.

Further elution with pentane/dichloromethane (2:8) and dichloromethane gave the diyne dialdehyde **16** together with the dimer of **13**, the butadiynyl dialdehyde **15**. To register the spectroscopic data of **14** and **15**, a small amount of this mixture was separated by repeated preparative thin layer chromatography with pentane/dichloromethane (25:75). Pure **16** is a colorless solid, m. p. 104-106 °C. - ^1H NMR (200 MHz, $CDCl_3$): δ = 2.65-3.35 (m, 12 H, 3.68-3.80 (m, 2 H), 4.10-4.21 (m, 2 H), 6.44-6.67 (m, 10 H, paracyclophanyl H), 7.37-7.42 (m, 2 H, phenyl H), 7.51 (d, *J* = 1.7 Hz, 2 H, paracyclophanyl H), 7.68-7.74 (m, 2 H, phenyl H), 9.91

ppm (two s, 2 H, 2 formyl H). - ^{13}C NMR (50 MHz, $CDCl_3$): δ = 33.3 (t), 33.9 (t), 34.1 (t), 34.4 (t), 92.5 (s), 92.6 (s), 92.8 (s), 92.8 (s), 124.8 (s), 124.8 (s), 125.3 (d), 125.6 (s), 125.6 (s), 127.8 (s), 128.2 (d), 128.2 (d), 129.0 (s), 132.5 (d), 132.9 (d), 133.9 (d), 133.9 (d), 134.9 (d), 135.2 (d), 135.9 (d), 136.2 (s), 136.3 (d), 136.4 (s), 138.3 (d), 138.3 (d), 139.9 (s), 140.5 (s), 140.6 (s), 142.4 (s), 142.4 (s), 142.5 (s), 192.6 ppm (d). - MS (EI): *m/z* (%) = 594 (40) [M^+], 462 (43), 329 (100), 313 (75). The pure dimer of 4-ethynyl-16-formyl-paracyclophane (**13**), dialdehyde **17**, has m. p. >220 °C (decomp.). - ^1H NMR (200 MHz, $CDCl_3$): δ = 2.83-3.46 (m, 12 H), 3.62-3.75 (m, 2 H), 4.15-4.26 (m, 2 H), 6.50-6.78 (m, 10 H), 7.50 (2×d, *J* = 1.8, 1.9 Hz, 2 H), 9.95 ppm (two s, 2 H, formyl). - ^{13}C NMR (50 MHz, $CDCl_3$)): δ = 33.5 (t), 34.0 (t), 34.3 (t), 77.9 (s), 82.0 (s), 123.4 (s), 133.6 (d), 134.1 (d), 135.7 (d), 135.9 (d), 136.4 (s), 136.8 (d), 138.2 (d), 140.2 (s), 140.4 (s), 142.3 (s), 143.6 (s), 192.8 ppm (d). - MS (EI): *m/z* (%) = 518 (40) [M^+], 386 (87), 252 (100).

The reaction of dialdehydes 16 and 17 with the Bestmann-Ohira reagent: The mixture of dialdehydes **16** and **17** (665 mg) was dissolved in dichloromethane (10 mL) and anhydrous methanol (30 mL) under nitrogen. Then

ca. 2 g of Cs_2CO_3 and excess Bestmann-Ohira reagent (0.5 mL) was added. The reaction mixture was vigorously stirred at room. temp. until the starting material had been consumed (TLC monitoring). After the usual work-up (see above) the mixture was column chromatographed and the mixture of the tetraacetylenes corresponding to **16** and **17** (total amount: 568 mg, ~85%) was separated by preparative TLC with pentane/dichloromethane (4:1) several times to yield the pure tetraacetylene corresponding to **17** (ca. 200 mg) as a colorless solid, m. p. 190-191 °C. - ^{1}H-NMR (200 MHz, $CDCl_3$): δ = 2.78-3.26 (m, 12 H), 3.37 (s, acetylene), 3.37 (s, acetylene), 3.57-3.74 (m, 4 H), 6.55 (m, 8 H), 7.14 ppm (m, 4 H). - ^{13}C NMR (50 MHz, $CDCl_3$): δ = 33.4 (t), 33.7 (t), 34.2 (t), 34.4 (t), 80.8 (d, acetylene), 82.1 (s, acetylene), 83.3 (s, acetylene), 123.5 (s), 123.7 (s), 133.5 (two d), 133.7 (d), 133.9 (d), 134.7 (d), 134.9 (d), 139.6 (s), 139.8 (s), 142.7 (s), 143.7 ppm (s).- IR (diamond-ATR): nu(tilde) = 3282 (m), 2927 (m), 2851 (m), 2205 (w), 2134 (w), 2099 (w), 1903 (w), 1585 (m), 1479 (m), (1449 (m), 1432 (m), 1403 (m), 895 (s), 872 (s), 719 (s), 648 (s), 604 cm^{-1} (s). - UV (EtOH): λ_{max} (lgε) = 232 (4.79), 298 (4.23), 316 (4.32), 336 (4.39), 364 nm (4.35). - MS (EI): *m/z* (%) = 510 (38) [M^+], 382

(43), 254 (75), 252 (100). - HRMS (EI): calcd. for $C_{40}H_{30}$ 510.2348; found 510.2333.

In a qualitative Glaser–Hay coupling experiment (see above) with 51 mg (0.1 mmol) of this hydrocarbon, a mixture of **11** and **12** was formed as shown by GC/MS analysis.

The tetraacetylene derived from **16**, the hydrocarbon **18**, (290 mg) is a colorless solid with m. p. 100-103 °C. - ^1H NMR (200 MHz, $CDCl_3$): δ = 2.68-3.27 (m, 12 H), 3.16 (s, acetylene), 3.21 (s, acetylene), 3.37-3.82 (m, 4 H), 6.51 (d, *J* = 0.9 Hz, 4 H, paracyclophanyl H), 6.53 (s, 4 H, paracyclophanyl H), 7.14 (d, *J* = 3.6 Hz, 2 H, paracyclophanyl H), 7.18 (d, *J* = 9.0 Hz, 2 H, paracyclophanyl H), 7.37 (dd, *J* = 3.4, 5.8 Hz, 2 H, phenyl), 7.70-7.74 ppm (dd, *J* = 3.4, 5.8 Hz, 2 H, phenyl). - ^{13}C NMR (50 MHz, $CDCl_3$): δ = 33.4 (t), 33.6 (t), 34.2 (t), 34.5 (t), 80.6 (d, acetylene), 83.4 (s, acetylene), 83.4 (s, acetylene), 92.1 (s, acetylene), 93.2 (s, acetylene), 123.7 (s), 124.9 (s), 125.9 (s), 127.9 (d), 132.4 (d), 133.1 (d), 133.4 (d), 133.5 (d), 134.1 (d), 134.1 (s), 134.7 (d), 139.6 (s), 139.7 (s), 142.5 (s), 142.6 ppm (s). - IR (diamond-ATR): nu(tilde) = 3287 (m), 2924 (m), 2212 (w), 2095 (w), 1978 (w), 1907 (w), 1483 (m), 1434 (m), 896 (s), 875 (s), 757

(s), 722 (s), 648 cm^{-1} (s). - UV (CH_2Cl_2): λ_{max} (lgε) = 229 (4.69), 301 nm (4.36). - MS (EI): *m/z* = 586 (67) [M^+], 427 (42), 326 (67), 313 (100). - HRMS (EI): Calcd. for $C_{46}H_{34}$ 586.2661; found 586.2653.

Glaser–Hay cyclization of 18, formation of 19 and 20: A solution of tetrayne **18** (35 mg, 0.06 mmol) in anhydrous dichloromethane (50 mL) was added to a mixture of CuCl (100 mg, 1 mmol) and TMEDA (0.6 mL). The mixture was stirred for 12 h under dry air ($CaCl_2$–tube). Water was added, the organic phase was washed with brine, dried with $MgSO_4$, and the solvent was evaporated to dryness. The resulting mixture was separated by repeated preparative TLC with pentane/dichloromethane (7:3). The separation afforded first the "crossed" isomer **20** (3 mg, 8.6%) as colorless crystals (from hexane/benzene), m. p. 230-250 °C (decomp.). - ^{1}H NMR (400 MHz, $CDCl_3$): δ = 2.72-2.80 (m, 2 H), 2.91-3.20 (m, 10 H), 3.50-3.64 (m, 4 H), 6.37 (dd, *J* = 1.7, 7.9 Hz, 2 H, paracyclophanyl H), 6.44 (d, *J* = 7.9 Hz, 2 H, paracyclophanyl H), 6.55 (d, *J* = 7.9 Hz, 2 H, paracyclophanyl H), 6.62 (dd, *J* = 1.8, 7.9 Hz, 2 H, paracyclophanyl H), 7.01 (d, *J* = 1.7 Hz, 2 H, paracyclophanyl H), 7.22 (dd, *J* = 3.4, 5.9 Hz, 2 H, phenyl H), 7.36 (d, *J* = 1.7 Hz, 2 H, paracyclophanyl H), 7.54 ppm (dd, *J* = 3.4, 5.8 Hz, 2 H, phenyl). - ^{13}C

NMR (100 MHz, $CDCl_3$): δ = 33.3 (t), 33.8 (t), 34.0 (t), 34.6 (t), 79.7 (s, acetylene), 85.2 (acetylene), 92.0 (s, acetylene), 94.2 (s, acetylene), 124.0 (s), 124.6 (s), 124.8 (s), 127.7 (d), 131.9 (d), 133.2 (d), 133.3 (d), 133.3 (d), 134.2 (d), 135.6 (d), 138.1 (d), 139.1 (s), 140.1 (s), 142.3 (s), 143.1 ppm (s). - IR (diamond-ATR): nu(tilde) = 2925 (m), 2851 (w), 2190 (w), 1586 (w), 1450 (m) 1433 (w), 1433 (m), 1404 (w), 759 (m), 722 (m), 676 (s), 652 cm^{-1} (s). - UV (CH_2Cl_2): λ_{max} (lgε) = 229 (4.77), 255 (4.67), 297 (4.48), 318 nm (4.49). - MS(EI): *m/z* (%) = 584 (100)[M^+], 328 (55), 313 (65). - HRMS (EI). calcd. for $C_{46}H_{32}$ 584.2504; found 584.2501.

As a second fraction the separation afforded the " parallel" isomer **19** (24 mg, 68%) as colorless crystals (from pentane/dichloromethane), m.p. 220-240 °C (decomp.). - ^{1}H NMR (400 MHz, $CDCl_3$): δ = 2.85-2.92 (m, 2 H), 2.95-3.00 (m, 2 H), 3.01-3.33 (m, 8 H), 3.66-3.78 (m, 4 H), 6.50 (dd, *J* = 1.4, 1.7, 8.1 Hz, 2 H, paracyclophanyl H), 6.52 (d, *J* = 7.9 Hz, 2 H, paracyclophanyl H), 6.60 (d, *J* = 7.9 Hz, 2 H, paracyclophanyl H), 6.65 (dd, *J* = 1.7, 7.9 Hz, 2 H, paracyclophanyl H), 7.10 (d, *J* = 1.7 Hz, 2 H, paracyclophanyl H), 7.36 (dd, *J* = 3.3, 5.8 Hz, 2 H, phenyl H), 7.49 (d, *J* = 1.1 Hz, 2 H, paracyclophanyl

H), 7.67 ppm (dd, J = 3.4, 5.8 Hz, 2 H, phenyl H). - ^{13}C NMR (100 MHz, $CDCl_3$): δ = 33.6 (t), 33.9 (t), 34.1 (t), 34.5 (t), 79.3 (s, acetylene), 84.9 (s, acetylene), 92.1 (s, acetylene), 93.3 (s, acetylene), 124.4 (s), 124.6 (s), 125.6 (s), 127.7 (d), 132.6 (d), 133.1 (d), 133.3 (d), 133.6 (d), 133.7 (d), 136.3 (d), 139.2 (s), 140.0 (s), 142.2 (s), 143.3 ppm (s). - IR (diamond-ATR): nu(tilde)= 2928 (m), 2195 (w), 1587 (m), 1488 (m), 1436 (m), 897 (m), 875 (m), 744 (s), 729 (s), 702 (s), 649 cm^{-1} (s). - UV (CH_2Cl_2): λ_{max} (lgε) = 229 (4.66), 255 (4.54), 295 (4.54), 339 (4.68), 362 nm (4.53). - MS (EI): m/z (%) = 584 (40) [M^+], 313 (10), 295 (10), 221 (25). - HRMS (EI): calcd. for $C_{46}H_{32}$: 584.2504; found 584.2500.

The separation also afforded a small amount of the starting material **18** (5 mg, 14 %).

Glaser-Hay coupling of 4,16-diethynyl[2.2]paracyclophane (4) with 1,2-diethynylbenzene (1): To dichloromethane (1.2 L) was added CuCl (5 g, 50 mmol) and TMEDA (5.8 g, 50 mmol, ca. 7.5 mL), and the solution was stirred for 15 min under air. To this solution, a solution of 4,16-diethynyl[2.2]paracyclophane (**4**, 128 mg, 0.5 mmol) and 1,2-diethynylbenzene (**1**, 70 mg, 0.55 mmol) in dichloromethane (250

mL) was slowly added over 24 h. The reaction mixture was quenched with water and thrice washed with water, dried ($MgSO_4$), and evaporated to dryness in vacuo. The crude mixture was chromatographed on silica gel (25 g). Elution with pentane followed by pentane/dichloromethane (95:5) afforded a yellow solid (40 mg, 32%) of the dimer of **1**, the known hydrocarbon **21** [12]. Further elution with pentane/dichloromethane (90:10 and later 80:20) afforded the heterodimer **22** as pale yellow crystals which darkened quickly at room. temp. (85 mg, 45%); on heating, these turned black rapidly and decomposition set in between 115-140 °C. - ^1H NMR (400 MHz, $CDCl_3$): δ = 2.65-2.75 (m, 4 H), 3.04 (t, *J* = 10.9 Hz, 2 H), 3.21-3.34 (m, 2 H), 6.45 (dd, *J* = 1.7, 8.0 Hz, 2 H, paracyclophanyl H), 6.47 (d, *J* = 7.9 Hz, 2 H, paracyclophanyl H), 7.21 (d, *J* = 1.6 Hz, 2 H, paracyclophanyl H), 7.26 (dd, *J* = 3.3, 5.8 Hz, 2 H, phenyl), 7.38 ppm (dd, *J* = 3.3, 5.8 Hz, 2 H, phenyl). - ^{13}C NMR (100 MHz, $CDCl_3$): δ = 33.8 (t), 35.0 (t), 79.5 (s), 82.8 (s), 86.4 (s), 89.6 (s), 125.0 (s), 128.6 (d), 129.1 (d), 129.3 (s), 132.5 (d), 135.2 (d), 139.8 (s), 140.0 (d), 140.6 ppm (s). - IR (diamond-ATR): nu(tilde) = 2924 (m), 2850 (m), 2184 (m), 1900 (w), 1484 (m), 1461 (m), 1448 (m), 1432 (m), 1402 (m), 895 (m), 756 (s), 720 (s), 642 (s), 550 cm^{-1} (m). - UV

(CH_2Cl_2): λ_{max} (lgε) = 234 (4.94), 307 (4.39), 319 (4.30), 342 (4.18), 364 nm (3.69). - MS (EI): *m/z* (%) = 378 (100), 374 (88), 372 (30), 361 (28), 350 (15), 187 (43).

Sonogashira coupling of 4-(2-iodophenylethynyl)-16-formyl[2.2]paracyclophane (15) with TMS-acetylene: A degassed solution of aldehyde **15** (400 mg, 0.87 mmol) in a mixture of toluene (20 mL), triethylamine (10 mL) and TMS-acetylene (1.5 g) was prepared in a Schlenk tube. Tetrakistriphenylphosphine palladium(0) (80 mg, 0.07 mmol) and CuI (50 mg, 0.26 mmol) were added and the reaction mixture was stirred for 12 h at room. temp. To the reaction mixture was added a solution of dilute aqueous hydrochloric acid, then the phases were separated, and the aqueous phase was extracted once more with dichloromethane. The combined organic solutions were washed with water, sodium bicarbonate solution and brine, and dried ($MgSO_4$). The solvent was removed under reduced pressure and the crude mixture was column chromatographed on 30 g of silica gel. Elution with pentane/dichloromethane (1:1) afforded the solid TMS-compound **23** (360 mg, 68%). - ^{1}H NMR (200 MHz, $CDCl_3$): δ = 0.30 (s, 9 H), 2.78-3.21 (m, 4 H), 3.34-3.49 (m, 2 H), 3.80-3.92 (m, 2 H), 4.13-4.24 (m, 2 H), 6.45-6.70 (m, 5 H, paracyclophanyl H), 7.14-7.37 (m, 2 H, paracyclophanyl + phenyl H), 7.53-7.60 (m, 3 H, phenyl), 9.91 ppm (s, 1 H, formyl H). - ^{13}C NMR (50 MHz, $CDCl_3$): δ = 0.0017 (q, TMS), 33.2 (t), 32.8 (t), 33.9 (t), 34.2 (t), 92.4 (s), 92.8 (s),

98.4 (s), 103.9 (s), 124.8 (s), 125.1 (s), 125.8 (d), 127.8 (d), 128.2 (d), 132.2 (d), 132.7 (d), 133.1 (d), 133.8 (d), 134.3 (s), 135.6 (d), 136.3 (d), 138.23 (d), 139.9 (s), 140.4 (s), 142.2 (s), 142.2 (s), 192.7 ppm (d).

Preparation of triacetylene 24: A solution of **23** (360 mg, 0.83 mmol) in anhydrous dichloromethane (10 mL) and methanol (20 mL) was added to Cs_2CO_3 (1.5 g) and Bestmann-Ohira reagent (0.3 mL, 0.24 g, 1.3 mmol). The reaction mixture was stirred under nitrogen at room. temp. for 48 h. For work-up, water and dichloromethane were added and the phases were separated. The aqueous phase was extracted once more with dichloromethane. The combined organic solutions were washed with brine and dried ($MgSO_4$). After solvent removal in vacuo the mixture was chromatographed on silica gel (40 g) with pentane/dichloromethane (1:1) to afford triacetylene **22** (200 mg, 67%), as a colorless solid, m. p. 127 °C (decomp.). - ^{1}H NMR (200 MHz, $CDCl_3$): δ = 2.79-2.95 (m, 2 H), 3.02-3.40 (m, 4 H), 3.34 (s, 1 H, acetylene), 3.50 (s, 1 H, acetylene), 3.59-3.91 (m, 2 H), 6.57 (m, 4 H, paracyclophanyl H), 7.20 (s, 1 H, paracyclophanyl H), 7.26 (d, J = 1.5 Hz, 1 H, paracyclophanyl H), 7.29-7.44 (m, 2 H, phenyl), 7.61-7.66 ppm (m, 2 H, phenyl). - ^{13}C NMR (50 MHz, $CDCl_3$): δ = 33.4 (t), 33.5 (t), 34.3 (t), 34.5 (t), 80.6 (d), 81.3 (d), 82.7 (s), 83.7 (s),

91.3 (s), 93.3 (s), 123.7 (s), 124.3 (s), 124.6 (s), 127.0 (s), 127.8 (d), 128.7 (d), 128.6 (d), 132.2 (d), 132.8 (d), 133.4 (d), 133.6 (d), 133.7 (d), 134.1 (d), 134.8 (d), 139.7 (s), 139.8 (s), 142.6 (s), 142.8 ppm (s). - IR (diamond-ATR): nu(tilde) = 3296 (m), 3268 (s), 2955 (m), 2203 (w), 2099 (w), 1483 (m), 1404 (m), 1320 (m), 759 (s), 720 (s), 635 cm^{-1} (s). - UV (CH_2Cl_2): λ_{max} (lgε) = 232 (4.61), 252 (4.36), 314 nm (4.64). - MS (EI): *m/z* (%) = 356 (23)[M^+], 341 (25), 226 (100).

Cobalt complex 26: A solution of $CpCo(CO)_2$ (0.5 mL, 0.68 g, 3.1 mmol) in degassed *p*-xylene (8 mL) was added over 10 min to a solution of triacetylene **24** (200 mg, 0.56 mmol) in degassed *p*-xylene (45 mL). The reaction mixture was heated at reflux and simultaneously irradiated with a 500 W daylight lamp for 20 min. The mixture was cooled and chromatographed on silica gel (70 g) with pentane to remove the xylene, followed by elution with pentane/dichloromethane (7:3) to afford pure **26** (109 mg, 40%) as orange crystals (from pentane/dichloromethane), m. p. 120 °C. - ^{1}H NMR (400 MHz, $CDCl_3$): δ = 2.69 (m, 1 H), 2.80-2.89 (m, 2 H), 3.06-3.13 (m, 2 H), 3.27-3.36 (m, 2 H), 3.39-3.46 (m, 1 H), 4.36 (s, 1 H), 4.51 (s, 1 H), 4.61 (s, 5 H), 6.13 (d, *J* = 7.5 Hz, 1 H, phenyl H), 6.34 (d, *J* = 7.8 Hz, 1 H, phenyl H), 6.61 (s, 1 H, paracyclophanyl H), 6.63 (d, *J* = 8.2 Hz, 1 H, phenyl H), 6.79 (d, *J* =7.6 Hz, 1 H, phenyl H), 7.26 (d, *J* = 7.3 Hz, 1 H, paracyclophanyl H), 7.30 (d, *J* = 7.6 Hz, 1 H, paracyclophanyl H), 7.33 (s, 1 H, paracyclophanyl H), 7.43

(d, *J* = 7.0 Hz, 1 H, paracyclophanyl H), 7.83 ppm (d, *J* = 7.4 Hz, 1 H, paracyclophanyl H). - ^{13}C NMR (100 MHz, $CDCl_3$): δ = 32.1 (t), 32.8 (t), 34.0 (t), 35.0 (t), 59.0 (d), 61.9 (d), 75.9 (s), 80.8 (s), 81.0 (d), 96.4 (acetylene), 100.5 (s, acetylene), 119.6 (s), 125.1 (d), 126.8 (d), 127.5 (d), 129.9 (d), 130.6 (d), 131.5 (d), 131.7 (d), 131.8 (d), 132.3 (d), 134.4 (s), 136.0 (s), 138.5 (d), 138.6 (s), 139.5 (s), 140.2 (s), 143.8 ppm (s). - IR (diamond-ATR): nu(tilde) = 2915 (w), 2850 (w), 2203 (w), 1488 (m), 1450 (m), 1433 (m), 807 (m), 756 (s), 726 (s) cm^{-1}. - UV (CH_2Cl_2): λ_{max} (lgε) = 232 (4.58), 264 (4.42), 308 nm (4.40). - MS (EI): *m/z* (%) = 480 (100)[M^+], 353 (30), 339 (23), 339 (15). - HRMS (EI): calcd. for $C_{33}H_{25}Co$: 480.1288; found: 480.1281

X-Ray structure determinations

Crystals were mounted in inert oil on glass fibres and transferred to the cold gas stream of the diffractometer (Siemens P4 for **11**, Bruker SMART 1000 CCD for all others). Intensity data were recorded using monochromated Mo *Kα* radiation (λ = 0.71073 Å). No absorption corrections were implemented except for **26** (multi-scans). The structures were refined anisotropically on F^2 using the program SHELXL-97 [2].

Hydrogen atoms were refined using a riding model starting from calculated positions. Compounds **11** and

22 are not pure enantiomers and crystallize only by chance in Sohncke space groups. For these structures and also for **20**, the anomalous scattering is not significant; Friedel opposite reflections were therefore merged and the Flack parameters are thus meaningless. Compound **19** crystallizes as a dichloromethane monosolvate; the asymmetric unit of compound **20** contains three molecules of **20** and four benzene molecules. All solvent molecules are ordered. Because **20** is a large (162 carbons in the asymmetric unit) and non-centrosymmetric structure, the data are somewhat weak and limited in number; an extensive system of restraints to the displacement parameters (DELU/SIMU) was used to improve refinement stability. Formal molecular symmetries were determined using the program (PLATON) [3].

The standard cyclophane numbering is retained for structures **11** and **12**, for which the asymmetric unit consists of only half a molecule, but not for the other structures, which are appreciably more complex. For individual molecular dimensions, see the crystallographic data, which have been deposited with the Cambridge Crystallographic Data Centre as supplementary publications no. CCDC-984749 (**11**), -984750 (**12**), -984751 (**19**), -984752 (**20**), -984753

(**22**), -984754 (**26**). Copies of the data can be obtained free of charge from www.ccdc.cam.ac.uk/data_request/cif.

Compound	**11**	**12**	**19·CH$_2$Cl$_2$**	**20·4/3 C$_6$H$_6$**	**22**	**26**
Formula	$C_{40}H_{28}$	$C_{40}H_{28}$	$C_{47}H_{34}Cl_2$	$C_{54}H_{40}$	$C_{30}H_{18}$	$C_{33}H_{25}Co$
M_r	508.62	508.62	669.64	688.86	378.44	480.46
Habit	colourless tablet	pale yellow needle	colourless tablet	colourless tablet	colourless prism	pale brown tablet
Cryst. size (mm)	0.5 × 0.3 × 0.25	0.16 × 0.09 × 0.08	0.35 × 0.25 × 0.12	0.4 × 0.4 × 0.2	0.45 × 0.16 × 0.16	0.3 × 0.2 × 0.06
Crystal system	orthorhombic	Monoclinic	monoclinic	orthorhombic	monoclinic	monoclinic
Space group	$P2_12_12$	$P2_1/n$	$P2_1/n$	$Pna2_1$	$P2_1$	$P2_1/c$
Temperature (°C)	–100	–173	–140	–140	–140	–140
Cell constants:						
a (Å)	12.702(2)	7.5955(6)	13.0302(14)	24.026(2)	7.1059(10)	10.9186(11)
b (Å)	13.163(2)	13.9205(11)	12.9188(14)	35.986(4)	12.761(2)	14.4704(14)
c (Å)	8.0527(18)	12.2555(10)	21.200(2)	13.4324(13)	11.585(2)	14.2762(14)
α (°)	90	90	90	90	90	90
β (°)	90	90.580(2)	105.151(4)	90	106.128(5)	90.858(3)
γ (°)	90	90	90	90	90	90
V (Å^3)	1346.4	1295.75	3428.3	11613	1009.2	2255.3
Z	2	4	4	12	2	4
D_x (Mg m^{-3})	1.255	1.304	1.297	1.182	1.245	1.415
μ (mm^{-1})	0.07	0.07	0.22	0.07	0.07	0.78
F(000)	536	536	1400	4368	396	1000
$2\theta_{max}$	55	58.6	60	52.7	60	60
Refl. measured	2300	9433	37220	101957	11561	34080
Refl. indep.	1777	3203	9953	12409	3056	6606
R_{int}	0.074	0.041	0.040	0.154	0.082	0.069
Parameters	181	181	442	1459	271	307
Restraints	52	0	0	1528	1	0

$wR(F^2$, all refl.)	0.086	0.156	0.135	0.128	0.101	0.108
$R(F$, >4s(F))	0.043	0.058	0.047	0.052	0.042	0.043
S	0.84	0.99	1.05	1.03	0.99	0.98
max. $\Delta\rho$ (e Å^{-3})	0.14	0.39	0.87	0.21	0.27	0.52

Table S1. Crystallographic data of **11**, **12**, **19**, **20**, **22**, and **26**.

References

[1] L. Bondarenko, I. Dix, H. Hinrichs, H. Hopf, *Synthesis*, **2004**, 2751-2759.

[2] G. M. Sheldrick, *Acta Cryst*. **2008**, *A64*, 112–122.

[3] PLATON: A. L. Spek, University of Utrecht, Netherlands.
